# Spring and summer microhabitat use by Schlegel’s Japanese gecko, *Gekko japonicus* (Reptilia: Squamata: Gekkonidae), in urban areas

**DOI:** 10.1080/19768354.2018.1554542

**Published:** 2018-12-04

**Authors:** Dae-In Kim, Il-Kook Park, Jong-Sun Kim, Hidetoshi Ota, Woo-Jin Choi, Il-Hun Kim, Daesik Park

**Affiliations:** a Department of Biology, Kangwon National University, Chuncheon, South Korea; b Division of Science Education, Kangwon National University, Chuncheon, South Korea; c Institute of Natural and Environmental Sciences, University of Hyogo, Hyogo, Japan; d Department of Ecology and Conservation, National Marine Biodiversity Institute of Korea, Seochun, South Korea

**Keywords:** Nocturnal gecko, microhabitat, quantitative measure variable, season, *Gekko japonicus*

## Abstract

Differential microhabitat use may be beneficial to achieving fitness in seasonally variable environmental conditions. To explore whether the microhabitat use of the nocturnal Schlegel’s Japanese gecko, *Gekko japonicus*, varies seasonally and depends on juvenile, male, and female reproductive groups, we investigated five categorical and five quantitative measure variables of microhabitat use in a wild population both in spring and summer. Most geckos were found on white, vertical planes of concrete and plastered brick walls. None of the categorical variables (type of location, substrate, substrate color, light source, and refuge) significantly differed according to season or group, while substrate temperature and irradiance at the location where geckos were observed and the distance from the nearest potential refuge were significantly greater in summer than in spring. The quantitative measure variables did not differ among the reproductive groups. These results suggest that *G. japonicus* seasonally adjusts its microhabitat use mainly in terms of quantitative measure variables rather than categorical variables.

## Introduction

The use of appropriate microhabitats by animals may increase their fitness in terms of predator avoidance, efficient feeding, successful breeding, and environmental stress tolerance (Anderson 2007). In ectothermic animals, such as reptiles, body temperature maintenance is closely linked to physiological and behavioral performance efficiency (Angilletta et al. 1999). Body temperature can substantially affect daily and seasonal activity patterns, including microhabitat use, especially in nocturnal lizards (Huey et al. 1989; Hitchcock and McBrayer 2006). In general, air temperature has high seasonal variation, and differences in temperature should elicit changes in the body temperatures of nocturnal geckos (Kearney and Predavec 2000). Such changes may force geckos to adjust their activities in seasonally variable environmental conditions to achieve appropriate fitness. Although many studies on seasonally adjusted thermoregulation in nocturnal geckos have been performed (Huey et al. 1989; Hitchcock and McBrayer 2006), how these reptiles adjust their microhabitat use at night across different seasons is not well understood.

Schlegel’s Japanese gecko (*Gekko japonicus*) is a small nocturnal lizard found throughout eastern China, southern Korea, and most of Japan (Zhao and Adler 1993; Ota & Tanaka 1996; Lee et al. 2004). This species is mainly found in residential areas such as buildings and city parks in urban or suburban areas. *Gekko japonicus* predominantly prey on invertebrates such as various insects and spiders, which gather around light sources, by utilizing sit-and-wait hunting tactics (Werner et al. 1997). Studies of *G. japonicus* have mainly been conducted in China and Japan and have reported that sexual size dimorphism is not distinctive and that females oviposit between April and early August, while males develop sperm throughout the year (Ji et al. 1991). In Korea, selection for warm and un-disturbed shelters by *G. japonicus* was recently studied in a vivarium (Park et al. 2018). *Gekko japonicus* is maximally active at approximately 1–2 h after sunset but is sometimes active for longer periods (Tawa et al. 2014).

In both field and laboratory conditions, the activity and thermoregulatory performance of *G. japonicus* are low under low air temperature, reflecting the temperature dependency of the species (Hu and Du 2007; Tawa et al. 2014). In the field, the skin surface temperature of *G. japonicus* was approximately 25°C in spring and increased to 28–29°C in summer (Hu and Du 2007). Considering the different body temperatures of *G. japonicus* between spring and summer, they may also adjust their microhabitat use to achieve appropriate fitness in seasonally variable environmental conditions. *Gekko japonicus* is an appropriate species with which to test such a possibility in nocturnal lizards because these lizards are easily detectible near light sources at night and their habitats are often within small sections of urban or suburban areas (Werner et al. 1997). In addition, microhabitat use may differ according to juvenile, male, and female reproductive groups, possibly due to competition, by controlling body temperature or capturing prey, as is known in *Hemidactylus turcicus* (Saenz 1996; Williams and McBrayer 2007). Regardless, relevant studies have not been conducted in this species.

To explore whether microhabitat use by *G. japonicus* varies seasonally and depends on juvenile, male, and female reproductive groups, we investigated five categorical and five quantitative measure variables of microhabitat use in a wild population both in spring and summer.

## Materials and methods

### Study site and field investigation

**Figure 1.**
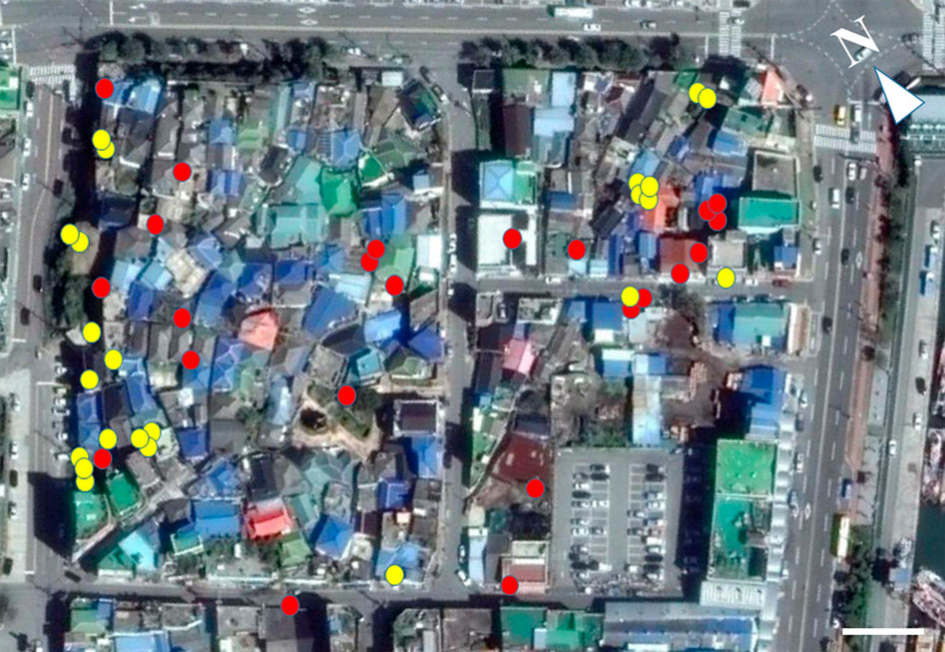
Photographs of the study site (34.8001923 N, 126.3895584 E, 210 m × 150 m) located in Dongmyeong-dong, Mokpo-si, Chonnam, South Korea, as obtained using Google Earth Pro. The locations where geckos were found are indicated by yellow for spring and red for summer. White bar = 20 m.

Our study site, which was approximately 400 m from a major harbor, was the small old town section (210 m × 150 m) of Dongmyeong-dong, Mokpo-si, Chonnam, South Korea (34.8001923 N, 126.3895584 E), and consisted of residential houses and small stores (Figure 1). Some trees were present along the street, and some vegetation existed in home gardens. We investigated the population both in spring (25 April 2017) and summer (17 August 2017). Our investigations started approximately after sunset and lasted 2–3 h (19:00–22:00). During the spring investigation, the mean air temperature ranged from 16.5–18.1°C (mean ± SE: 17.3 ± 0.36°C); in summer, it ranged from 25.5–27.4°C (mean ± SE: 26.2 ± 0.43°C). The temperature was measured at the Mokpo meteorological center (34.490172 N, 126.225186 E), which is located 3.1 km from the study site, and was obtained through the weather data opening portal (https://data.kma.go.kr/cmmn/main.do). We mainly searched for geckos on the walls of fences and buildings and on the planes of other structures such as electric poles, exhibition boards, and embankments, while slowly walking along the edges of roads within the site.

When a gecko was found, we recorded the GPS point (Oregon 550, Garmin Ltd. USA), date, and time. We then captured the gecko and determined whether it belonged to the juvenile reproductive group (approximately < 45 mm snout-vent length (SVL)) or the adult reproductive group (approximately > 50 mm SVL). We determined the sex of adults based on the relative size of the cloacal spurs (Tokunaga 1984). To prevent duplicate sampling of geckos, we kept the collected geckos in a box until the end of the daily investigation and then released them into the areas where the geckos were initially found.

For microhabitat use, we measured five quantitative measure and five categorical microhabitat variables based on previous studies (Gomez-Zlatar et al. 2006; Williams and McBrayer 2007). Considering the similar habitat location of our study population, which is within a small section of an urban area, we suspected that variables measured in previous studies could reveal important aspects of microhabitat use in our study population. The five categorical variables included the type of location, substrate, substrate color, nearest light source, and nearest potential refuge. The observation location was classified as a plane (wall, ceiling, or object surface), a crevice (or crack), a gap between two objects, or a street lamp. The substrate was classified as either concrete or plastered brick, rock, a concrete and rock mixture, or other materials such as wood, plastic and iron. Substrate color was recorded as white, gray, brown, blue, reddish, green, or yellow, and refuge type was recorded as a crevice or crack in rock, brick, concrete, eaves, a gutter, etc., vegetation (various plants, including ivy), or behind various objects such as vases, boxes, and other artificial structures. The light source illuminating the observation location was classified as either a white street lamp, yellow street lamp, house, or vending machine.

The five quantitative measure variables included the substrate temperature where a gecko was found, the irradiance on the location, the shortest distances to the nearest light source and the nearest potential refuge, and the shortest height from the ground. The substrate temperature of the exact site where a gecko was located was measured to the nearest 0.1°C using an infrared thermometer (AR-320, Smart Sensor Inc., Hong Kong). We measured the temperatures within 1 m from the site (62 of 64 cases), except two cases, which were measured at approximately 3 m from the site. Due to possible disturbance from infrared light, we did not directly measure the skin surface temperature of the gecko. In the field and the laboratory, the skin temperature of *G. japonicus* has been reported to be very similar to the temperature of its substrate (Hu and Du 2007); therefore, we used substrate temperature to represent body temperature. To record irradiance at the location of observation, we placed a digital illuminometer (TES-1337, TES, China) along the body axis of the gecko from the head to the tail on the same plane and measured the intensity in units of 0.01 Lux. Additionally, we measured the shortest distance to the nearest light source that primarily illuminated the geckos, the shortest distance to the nearest potential refuge, and the shortest height from the ground (or bottom plate) to the nearest mm using a laser distance meter (Fluke 414D, Fluke Korea, South Korea).

### Data analysis

For data analysis, the time at which each gecko was found was converted into the time after local sunset at the study site on the date of the investigation (www.timeanddate.com). To increase the central tendency of the data, we log-transformed (log_10_) all the quantitative measure variables, including the time after sunset. Even after transformation, the variables did not pass the normality test (Kolmogorov–Smirnov test, *P* < 0.05); therefore, nonparametric statistical analyses were applied when necessary.

The relationships between the time after sunset and the five quantitative measure variables were analyzed using a Spearman correlation test for both spring and summer. The recorded frequencies of categorical variables were compared using the Chi-square test (Preacher 2001) between spring and summer and among the juvenile, male, and female reproductive groups.

Differences in the five quantitative measure variables were initially analyzed using a multivariate general linear model. In the analysis, season and reproductive groups showed significant interactions for some quantitative measure habitat variables (Irradiance, *P *= 0.029; Distance to light source, *P *= 0.008); therefore, we separately analyzed the season and reproductive group effects on the use of microhabitats. For the independent variable Season, we used the reproductive group and the time after sunset as covariates, while for the reproductive group, we used the season and the time after sunset as covariates. In both analyses, dependent variables were the five quantitative measure habitat variables.

The data were presented as the mean ±1 SE. Except for on-line Chi-square tests, all the analyses were performed using SPSS (ver. 18.0).

## Results

### General trends

**Figure 2.**
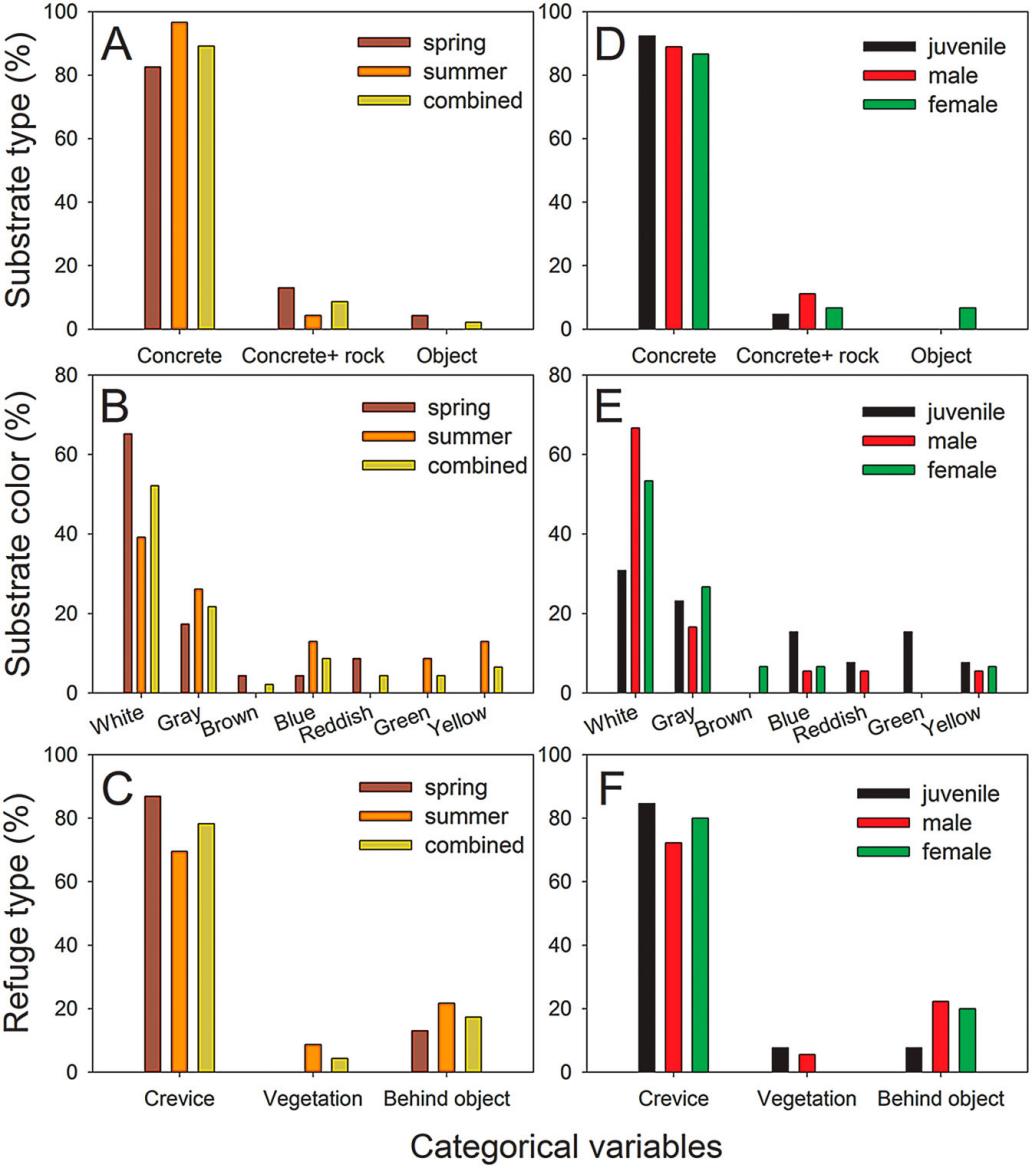
Comparison of the microhabitat use of *G. japonicus* in terms of categorical variables between spring and summer (a, b, c) and among different reproductive groups (d, e, f). None of the variables showed significant differences between the groups. ‘Combined’ in panels A, B, and C indicates the combined data from two seasons.

In the analyses, a total of 46 samples, including 23 samples each in spring (5 juveniles, 7 males, and 11 females) and summer (8 juveniles, 11 males, and 4 females), were used. Geckos were found in more diverse areas of the study site in summer than in spring (Figure 1). Unlike the quantitative measure variables, none of the categorical variables significantly differed among seasons and reproductive groups (see below). Therefore, we also combined the data of the two seasons for analysis to describe the general trends of microhabitat use in terms of the categorical variables (Figure 2(a–c)).

All 46 geckos were found on vertical planes, mainly on concrete or plastered brick substrates (41 cases, Figure 2(a)). Four geckos were found on a concrete + rock substrate, and one was found on an electric pole. In total, 24 and 10 geckos were found on white and gray substrates, respectively. In addition, we found four geckos on blue substrates and three on yellow substrates (Figure 2(b)). The remaining five geckos were found on brown (1 case), reddish (2 cases), and green (2 cases) substrates. As a potential refuge, most geckos used crevices (36 cases, Figure 2(c)), whereas eight and two geckos sought refuge behind objects and in vegetation, respectively. The light sources illuminating the geckos included white (44 cases) and yellow (2 cases) street lamps.

### Comparison between spring and summer

In spring, the distance to the light source (*P* < 0.001) was positively correlated with the time after sunset (*P* < 0.001), while the height from the ground was negatively correlated with the time after sunset (*P* = 0.027, Table 1). Additionally, the substrate temperature was negatively correlated with the distance to the refuge (*P* = 0.010). In summer, the time after sunset was not significantly correlated with any of the variables considered (*P* > 0.135, Table 1). Substrate temperature was positively correlated with the distance to the refuge (*P* = 0.044). In addition, irradiance was negatively correlated with the distance to the light source (*P* = 0.013).

**Table 1.** Correlations among time after sunset (TAS) and five quantitative measure microhabitat variables for *G. japonicus* in spring and summer. Significant correlations are indicated in bold.

	Spring (*n* = 23)					Summer (*n* = 23)				
	TAS	ST	ID	DL	DR	TAS	ST	ID	DL	DR
Substrate temperature (ST)	*r* = 0.008 *P* = 0.971					*r* = −0.321 *P* = 0.135				
Irradiance (ID)	*r* = 0.088 *P* = 0.691	*r* = 0.368 *P* = 0.084				*r* = 0.147 *P* = 0.502	*r* = 0.033 *P* = 0.881			
Distance from light source (DL)	***r* = 0.691** ***P* < 0.001**	*r* = 0.139 *P* = 0.528	*r* = 0.049 *P* = 0.825			*r* = −0.196 *P* = 0.370	*r* = −0.026 *P* = 0.907	***r* = −0.510** ***P* = 0.013**		
Distance from refuge (DR)	*r* = −0.103 *P* = 0.641	***r* = 0.527** ***P* = 0.010**	*r* = 0.352 *P* = 0.099	*r* = −0.135 *P* = 0.538		*r* = −0.081 *P* = 0.713	***r* = 0.424** ***P* = 0.044**	*r* = 0.050 *P* = 0.821	*r* = 0.046 *P* = 0.834	
Height	***r* = −0.461** ***P* = 0.027**	*r* = 0.154 *P* = 0.483	*r* = −0.138 *P* = 0.529	*r* = 0.072 *P* = 0.743	*r* = 0.214 *P* = 0.326	*r* = −0.062 *P* = 0.778	*r* = −0.279 *P* = 0.197	*r* = 0.102 *P* = 0.644	*r* = −0.210 *P* = 0.335	*r* = −0.088 *P* = 0.690

Although some differences in the use of substrate color and refuge types were identified, the five categorical variables were not significantly different between spring and summer (*P* > 0.092, Figure 2(a–c)).

**Figure 3.**
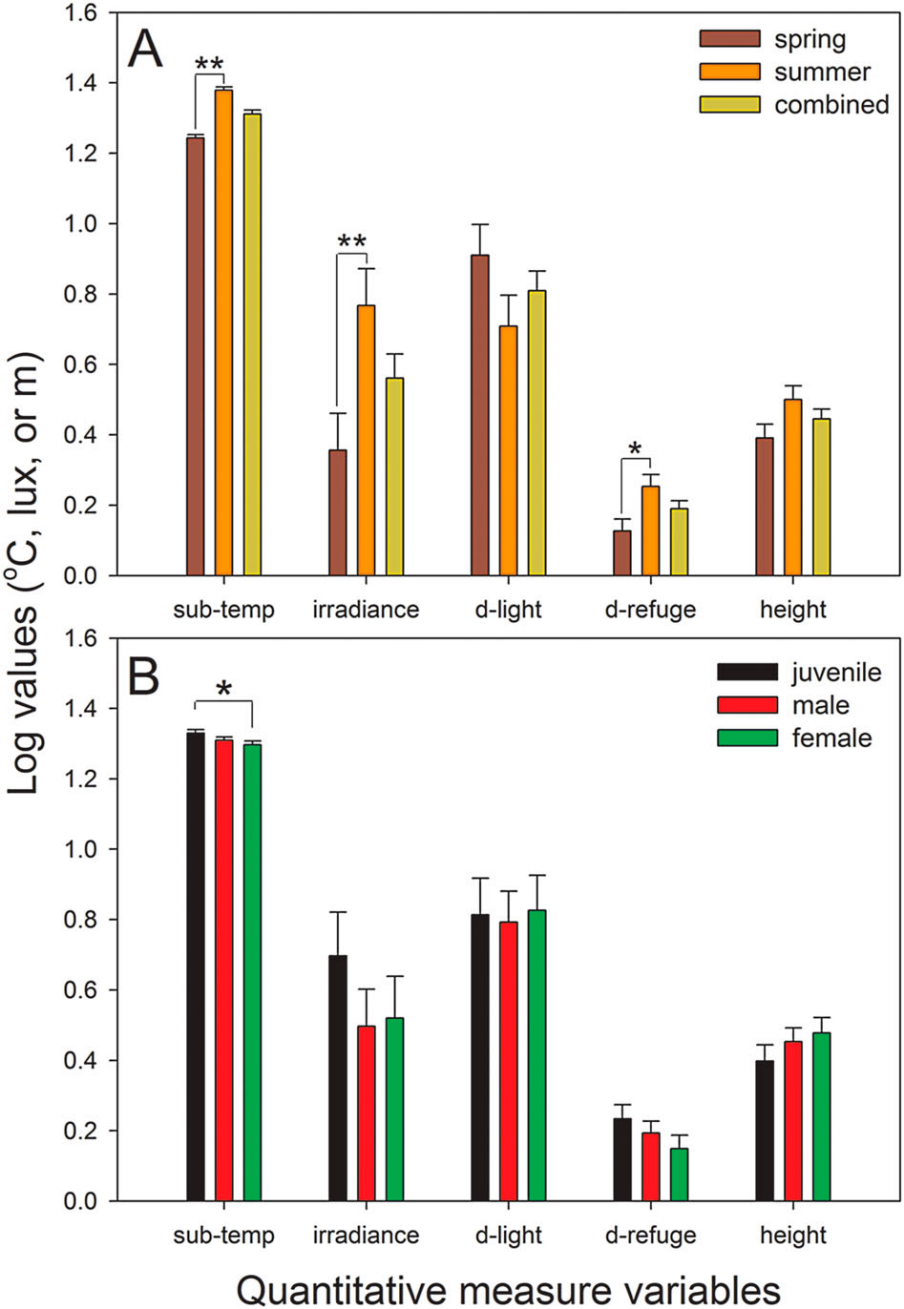
Comparison of the microhabitat use of *G. japonicus* in terms of quantitative measure variables between spring and summer (a) and among different reproductive groups (b). ‘Combined’ in panel A indicates the combined data from two seasons. The data are the estimated marginal means +1 SE, which considered covariates in the analysis model. Sub-temp, substrate temperature where geckos were observed; *d*-light, distance to the main light source illuminating the geckos; *d*-refuge, distance to the nearest potential refuge. *P < 0.05; **P < 0.01.

Unlike the categorical variables, three quantitative measure variables significantly differed between seasons. Substrate temperature and irradiance were higher (*F*_1, 46 _= 88.46, *P* < 0.001; *F*_1, 64_ = 6.19, *P* = 0.017, respectively, Figure 3(a)) and the distance to the refuge was greater (*F*_1, 64 _= 5.46, *P* = 0.024, Figure 3(a)) in summer than in spring. However, the distance to the light source (*P* = 0.155) and the height from the ground (*P* = 0.087) were not seasonally different.

### Comparison among the juvenile, male, and female reproductive groups

For the categorical variables, although juveniles tended to use more substrate colors than adults, none of the variables significantly differed among the reproductive groups (*P *> 0.491, Figure 2(d–f)).

Among the quantitative measure variables, only substrate temperature tended to be significant among the groups (*F*_2, 64 _= 2.67, *P* = 0.082). In a *post hoc* test, the substrate temperature of juveniles was higher than that of females (*P* = 0.028), but other comparisons were not significant (*P* > 0.05). The four remaining quantitative measure variables did not significantly differ among the groups (*P* > 0.326, Figure 3(b)).

## Discussion

### General trends

*G. japonicus* seems to prefer microhabitats where it can avoid predators and easily capture prey. Most geckos were found on the vertical planes of walls composed of concrete or plastered brick. These flat areas are known to be suitable for feeding on insects while being difficult to access by predators (Punzo 2001). *Gekko japonicus* individuals were largely found on the white substrates that apparently effectively attract insects. Nocturnal geckos mainly feed on insects that gather near light sources (Saenz 1996; Werner et al. 1997). Additionally, *G. japonicus* individuals were also found on gray substrates, which may provide camouflage and attract certain insects (Gomez-Zlatar et al. 2006). Crevices or cracks were the most frequently identified refuge type in the study site, which can provide safety because geckos can flatten their bodies to fit inside crevices (Caldwell et al. 2014). When available, vegetation also appeared to be a possible refuge type, but this refuge type was not frequently recorded because vegetation areas were limited in the study site. White street lamps were the most frequently selected light source, but whether this was due to the large number of white street lamps at the study site or reflected a direct preference is unclear.

### Comparison between spring and summer

Unlike the quantitative measure variables, the categorical microhabitat variables were not different between seasons. Our result is consistent with a recent finding in *Psammodromus algirus* diurnal lizards (Díaz et al. 2005), whereby microhabitat selection was not affected by a seasonally altered thermal environment, suggesting that other factors such as availability of microhabitat types might be important. Two possible explanations may account for our result. First, the results may have been affected by the limited diversity of the microhabitat in the urban areas that we investigated. We could not enter private houses and buildings and therefore searched for geckos mainly along the edges of small roads. Additionally, we investigated mostly feeding or mating microhabitat use because our investigation did not include a daytime search. Due to such a limitation, we may not have fully encompassed the various microhabitat types that geckos can use in different seasons, resulting in statistically non-significant results. Second, the results were more likely caused by geckos’ efficient adjustments of seasonal microhabitat use through changes in terms of quantitative measure variables. Seasonal shifts in quantitative measure microhabitat use have previously been implicated in *Conolophus pallidus* iguanas (Christian et al. 1983); however, to our knowledge, directly related studies have not been reported in nocturnal geckos. In summer, we generally observed geckos in more diverse areas of the site, but microhabitat use did not differ between seasons in terms of categorical variables (Figure 1). Therefore, since we only detected significant changes in quantitative measure microhabitat variables between seasons, we speculate that geckos can achieve appropriate fitness in summer mainly by adjusting the quantitative measure rather than categorical variables of the microhabitat. Microhabitat use by *G. japonicus* varied seasonally in terms of quantitative measure variables. This result may be largely caused by the different body temperatures of *G. japonicus* in spring and summer. In spring, *G. japonicus* likely experiences lower body temperatures because substrate temperatures are lower in spring than in summer. This relatively low temperature condition in spring may limit various activities, possibly including microhabitat use, in terms of quantitative measure variables. Considering that body temperature and mobility are highly correlated (Huey et al. 1989), low body temperature is thought to cause *G. japonicus* to remain closer to refuge sites in spring. In this study, the temperature of substrates where geckos were located showed a negative correlation with the distance from refuge sites. Subsequently, low irradiance tended to be related to greater distance from the light source and lower height from the ground in spring. In spring, temperature-dependent microhabitat use may be slightly amplified with the passage of time after sunset. The time after sunset was positively correlated with the distance to the light source but negatively correlated with the height from the ground. However, in summer, the time after sunset was not significantly correlated with any other quantitative measure variables. These discrepant results may be attributed to the declining pattern of air temperature after sunset in spring compared to that in summer. Our results suggest that *G. japonicus* actively adjusts its microhabitat use depending on body temperature, particularly in terms of quantitative measure variables.

### Comparison among the juvenile, male, and female reproductive groups

Juveniles tended to use white substrates less often, but used gray, blue, and green substrates more often than did male and female adults. Nevertheless, overall microhabitat use in terms of categorical variables did not differ significantly among the groups. As stated above, the limited diversity of the microhabitat investigated in this study and the importance of adjusting quantitative measure microhabitat variables may also be responsible for this result.

Unlike the categorical variables, substrate temperature tended to be different among the groups; specifically, the temperatures of juveniles were higher than those of females, which is consistent with a previous study of *G. japonicus* reporting that juveniles’ body temperatures were higher than those of adults (Hu and Du 2007). *Gekko japonicus* juveniles tended to exploit farther from refuge sites (see Figure 3(b)), though these findings were not statistically significant. These results may be explained by two factors. First, the higher body temperatures of juveniles may allow them to exploit more diverse and more vulnerable habitats due to enhanced performance following increased body temperature (Huey et al. 1989; Tawa et al. 2014). Second, adults may actively exclude juveniles from better feeding and safer habitats (e.g. Williams and McBrayer 2007). The precise factors responsible for such variable habitat use between juveniles and adults should be further explored and verified in the future.

The absence of differences in microhabitat use between males and females can be explained by a lack of distinctive sexual dimorphism (Tokunaga 1984). As previously reported in *H. turcicus* (Johnson et al. 2005), the known sexual dimorphism of *G. japonicus* may also be a simple byproduct of differential growth patterns rather than a direct fitness-related advantage, resulting in non-significant differences in microhabitat use between males and females. However, if time-series studies of a population are performed, females may still be found to prefer higher-temperature and more favorable feeding sites than males during a specific period of egg development.

## Conclusion

In this study, *G. japonicus* preferred microhabitats that enabled it to avoid potential predators but effectively acquire food sources. Seasonal and daily changes in air temperature, which is directly related to body temperature, had significant effects on the quantitative measure but not the categorical variables of microhabitat use of *G. japonicus*. In particular, during the summer, *G. japonicus* were found at locations with higher substrate temperature and greater irradiance and at a greater distance from the potential refuge than during the spring. Our results suggest that *G. japonicus* seasonally adjusts its microhabitat use mainly in terms of quantitative measure variables rather than categorical variables, possibly to achieve appropriate fitness in seasonally variable environmental conditions.

## Supplementary Material

Supp_Figure.docx
