# Supplementary material for: Spring and summer microhabitat use by Schlegel’s Japanese gecko, Gekko japonicus (Reptilia: Squamata: Gekkonidae), in urban areas
Source: Anim Cells Syst (Seoul). 2018 Dec 4;23(1):64–70. doi: 10.1080/19768354.2018.1554542 (PMC6394305; doi:10.1080/19768354.2018.1554542)

Supplemental Figure 1

**S Figure 1** Changes in the mean air temperature of the Mokpo city between 00:00 and 23:00 on April 25, 2017 (spring investigation date), and on August 17, 2017 (summer investigation date), measured approximately 3.1 km away from our study population. The data were obtained from the weather data opening portal (<https://data.kma.go.kr/cmmn/main.do)>. Arrows in the graph indicate the time of local sunset on the given day. Our investigation was conducted between 19:00 and 22:00 PM.


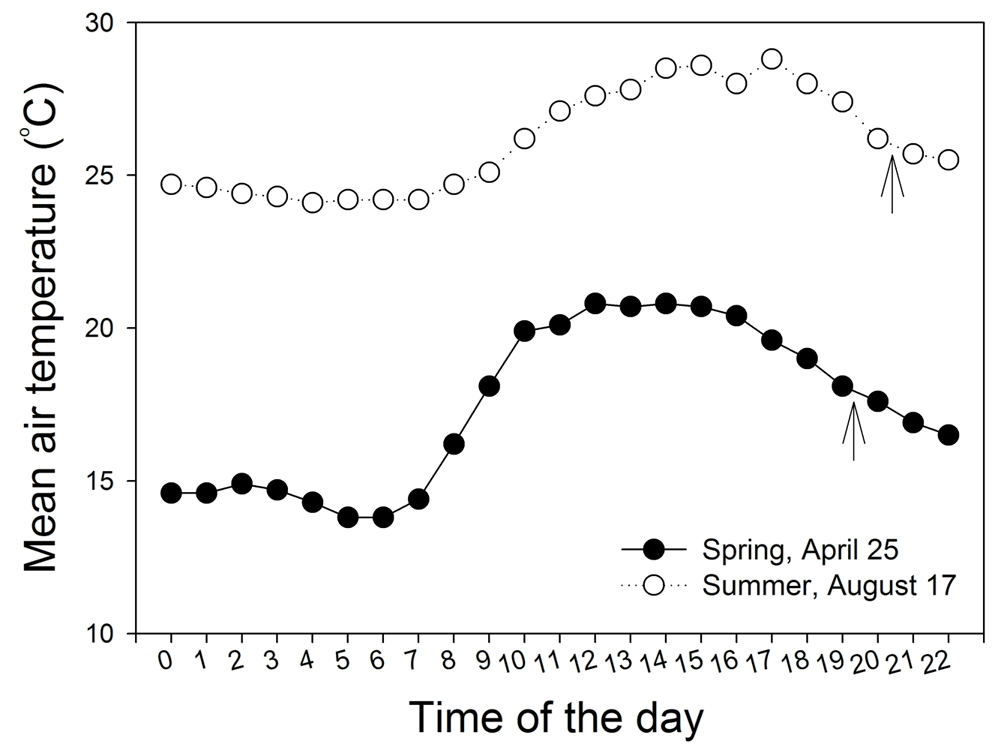

Supplement: Supp_Figure.docx [file TACS_A_1554542_SM9636.docx]
